# Erdosteine in children and adults with bronchiectasis (BETTER trial): study protocol for a multicentre, double-blind, randomised controlled trial

**DOI:** 10.1136/bmjresp-2023-002216

**Published:** 2024-05-07

**Authors:** Anne B Chang, Stephanie T Yerkovich, Katherine J Baines, Lucy Burr, Anita Champion, Mark D Chatfield, Kah P Eg, Vikas Goyal, Robyn L Marsh, Gabrielle B McCallum, Margaret McElrea, Steven McPhail, Lucy C Morgan, Peter S Morris, Anne M Nathan, Hannah O’Farrell, Marion O Sanchez, Marianne Parsons, André Schultz, Paul J Torzillo, Nicholas P West, Lesley Versteegh, Julie M Marchant, Keith Grimwood

**Affiliations:** 1 Department of Respiratory Medicine, Queensland Children's Hospital, South Brisbane, Queensland, Australia; 2 The Australian Centre for Health Services Innovation (AusHSI), Queensland University of Technology, Brisbane, Queensland, Australia; 3 Child and Maternal Health Division and andand NHMRC Centre for Research Excellence in Paediatric Bronchiectasis (AusBREATHE), Menzies School of Health Research, Darwin, Northern Territory, Australia; 4 School of Medicine and Public Health, The University of Newcastle, Newcastle, New South Wales, Australia; 5 Immune Health Research Program, Hunter Medical Research Institute, Newcastle, New South Wales, Australia; 6 Mater Health Services, South Brisbane, Queensland, Australia; 7 Department of Pharmacy, Queensland Children’s Hospital, Brisbane, Queensland, Australia; 8 The University of Queensland, Brisbane, Queensland, Australia; 9 Department of Paediatrics, Universiti Malaya Faculty of Medicine, Kuala Lumpur, Malaysia; 10 Department of Paediatrics, Gold Coast Health, Gold Coast, Queensland, Australia; 11 School of Health Sciences, University of Tasmania, Launceston, Tasmania, Australia; 12 School of Public Health and Social Work, Faculty of Health, Queensland University of Technology, Australian Centre for Health Services Innovation and Centre for Healthcare Transformation, Brisbane, Queensland, Australia; 13 Metro South Health, Clinical Informatics Directorate, Woollongabba, Queensland, Australia; 14 Department of Respiratory Medicine, Concord Repatriation General Hospital, Concord, New South Wales, Australia; 15 Section of Pediatric Pulmonology, Institute of Pulmonary Medicine, St. Luke's Medical Center, Quezon City, Philippines; 16 Parent Advisory Group, Cough, Asthma and Airways Group, Queensland University of Technology Faculty of Health, Kelvin Grove, Queensland, Australia; 17 Department of Respiratory Medicine, Princess Margaret Hospital for Children, Perth, Western Australia, Australia; 18 Wal-yan Respiratory Research Centre, Telethon Kids Institute & Division of Paediatrics, Faculty of Medicine, University of Western Australia, Perth, Western Australia, Australia; 19 Respiratory Medicine, Royal Prince Alfred Hospital, Camperdown, New South Wales, Australia; 20 Sydney Medical School, The University of Sydney Faculty of Medicine and Health, Sydney, New South Wales, Australia; 21 School of Medicine and Dentistry, Griffith University Griffith Health, Gold Coast, Queensland, Australia; 22 Departments of Infectious Disease and Paediatrics, Gold Coast Health, Gold Coast, Queensland, Australia

**Keywords:** Bronchiectasis, Paediatric Lung Disaese

## Abstract

**Introduction:**

Bronchiectasis is a worldwide chronic lung disorder where exacerbations are common. It affects people of all ages, but especially Indigenous populations in high-income nations. Despite being a major contributor to chronic lung disease, there are no licensed therapies for bronchiectasis and there remain relatively few randomised controlled trials (RCTs) conducted in children and adults. Our RCT will address some of these unmet needs by evaluating whether the novel mucoactive agent, erdosteine, has a therapeutic role in children and adults with bronchiectasis.

Our primary aim is to determine in children and adults aged 2–49 years with bronchiectasis whether regular erdosteine over a 12-month period reduces acute respiratory exacerbations compared with placebo. Our primary hypothesis is that people with bronchiectasis who regularly use erdosteine will have fewer exacerbations than those receiving placebo.

Our secondary aims are to determine the effect of the trial medications on quality of life (QoL) and other clinical outcomes (exacerbation duration, time-to-next exacerbation, hospitalisations, lung function, adverse events). We will also assess the cost-effectiveness of the intervention.

**Methods and analysis:**

We are undertaking an international multicentre, double-blind, placebo-RCT to evaluate whether 12 months of erdosteine is beneficial for children and adults with bronchiectasis. We will recruit 194 children and adults with bronchiectasis to a parallel, superiority RCT at eight sites across Australia, Malaysia and Philippines. Our primary endpoint is the rate of exacerbations over 12 months. Our main secondary outcomes are QoL, exacerbation duration, time-to-next exacerbation, hospitalisations and lung function.

**Ethics and dissemination:**

The Human Research Ethics Committees (HREC) of Children’s Health Queensland (for all Australian sites), University of Malaya Medical Centre (Malaysia) and St. Luke’s Medical Centre (Philippines) approved the study. We will publish the results and share the outcomes with the academic and medical community, funding and relevant patient organisations.

**Trial registration number:**

ACTRN12621000315819.

**WHAT IS ALREADY KNOWN ON THIS TOPIC**

- Bronchiectasis, ranking only behind asthma and chronic obstructive pulmonary disease as the most common chronic pulmonary disorder globally, is under-researched especially in children, with large knowledge gaps and no licensed therapies. Erdosteine, a novel oral mucoactive agent possessing additional anti-inflammatory and anti-microbial properties, may provide a clinical benefit.

**WHAT THIS STUDY ADDS**

- This is the first randomised controlled trial to combine children and adults with bronchiectasis into a single trial. It is also the first trial of erdosteine in people with bronchiectasis to inform whether this novel agent is well tolerated and reduces acute exacerbations and improves quality of life in this group of patients.

**HOW THIS STUDY MIGHT AFFECT RESEARCH, PRACTICE OR POLICY**

- If a clinical benefit is demonstrated, erdosteine could be easily incorporated into routine clinical practice, especially if well tolerated and cost-effective.

## Introduction

Bronchiectasis unrelated to cystic fibrosis (CF) is a chronic lung disorder affecting all age groups.^1^ It is characterised clinically by recurrent or persistent wet/productive cough, lower airway infection and inflammation, and radiographically by abnormal bronchial dilatation seen on chest CT scans.^1 2^ Acute exacerbations identified by new or worsening respiratory symptoms are also common and are important contributors to the overall burden of disease.^3^

The high prevalence of bronchiectasis among Indigenous children in high-income countries is well known. In remote Australian Indigenous communities of Central and Western Australia, bronchiectasis rates in children have remained stubbornly high at 1.5% during the last 20 years.^4 5^ In contrast, its burden among Indigenous children living in low-income and middle-income countries^6^ and non-Indigenous children is underappreciated,^7 8^ despite bronchiectasis ranking only behind asthma and chronic obstructive pulmonary disease (COPD) as the most common chronic pulmonary disorder worldwide^9^ resulting in substantial morbidity and mortality.^1 7^ Deemed by the European Respiratory Society (ERS) as one of the most neglected lung diseases in clinical practice,^10^ bronchiectasis, especially in children, is relatively under-researched^11^ and underserviced^12^ with large unmet needs.^13–15^ Consequently, critical, clinically relevant gaps remain,^13–15^ including the absence of licensed therapies for bronchiectasis and scarce data on acute respiratory exacerbations.^1^

Current guidelines^2 16 17^ for managing children and adults with bronchiectasis depict the limited availability of therapies specific to bronchiectasis. General therapies include nutritional care, additional vaccinations and pulmonary rehabilitation in adults while specific therapies comprise airway clearance techniques, antibiotics (for managing and preventing acute exacerbations^18 19^) and mucoactive agents (eg, hypertonic saline and mannitol). Without licensed therapies and high-level evidence, many current interventions used in people with bronchiectasis are generally extrapolated from studies and clinical experience gained from treating people with CF.^7^ However, given the very different pathobiological mechanisms between bronchiectasis and CF, it is of no surprise that some selected therapies, which are beneficial for CF, can be deleterious for people with bronchiectasis (eg, dornase alfa).^7^

The pathobiology of bronchiectasis includes the coexistence of chronic lower airway infection and inflammation, mucus hypersecretion and disruption of mucociliary clearance, which are all integral to the vicious vortex (previously labelled the vicious cycle) hypothesis of bronchiectasis,^7 20^ and are also thought to operate in people with COPD.^21^ Clinically these processes result in wet/productive cough, symptoms that are particularly bothersome for people with bronchiectasis.^14^

Recurrent respiratory exacerbations are also a characteristic feature of broncheictasis.^20 22^ Reducing exacerbations is important as these episodes impair quality of life (QoL) and can lead to an accelerated decline in lung function and high utilisation of healthcare resources.^20 22–24^ A prospective multicentre study involving 85 children with bronchiectasis found that the mean number of exacerbations was 3.3 (SD 2.2) per child-year.^25^ In any given month, 25% of these children were absent from school/childcare, 30% attended a healthcare appointment and 46% received antibiotics due to bronchiectasis while 11.4% of respiratory exacerbations required hospitalisation.^25^

Interventions that might reduce respiratory exacerbations and sputum volume would be beneficial to people with bronchiectasis as these factors are considered by clinicians and patients as the most challenging aspects of managing bronchiectasis.^13 14^ Mucoactive agents, such as erdosteine, are one such intervention that could address both of these patient and clinician priorities.^26–28^

Erdosteine is a novel thiol derivative, synthesised to overcome problems (eg, non-efficacy and toxicity related to increased free thiols in the circulation) observed with other thiols (eg, N-acetylcysteine).^21^ It is licensed currently in 40 countries, including the UK (it has orphan drug status in the USA,^29^ but not in Australia.^29^ Erdosteine is administered orally and has four main properties that could moderate the effects of ciliary dysfunction in the lungs (and thus potentially reduce exacerbations and improve QoL in people with bronchiectasis. These are as follows: (1) a mucoactive agent, modulating mucus production and viscosity, and increasing mucociliary transport; (2) an antioxidant; (3) airway anti-inflammatory and (4) bacterial antiadherence properties.^21 30^ Erdosteine has been used for short-term studies of acute respiratory infections in children (aged >1 year)^31^ and shown to be safe in these studies, which collectively involved approximately 500 children.^31–33^

The syndrome of chronic bronchitis (ie, chronic productive cough) is a component of bronchiectasis; in both conditions, mucus hypersecretion and dysfunctional mucociliary clearance are present.^1 34^ In a meta-analysis of 10 randomised controlled trials (RCTs), erdosteine (vs placebo) improved the clinical outcomes of adults with either COPD or chronic bronchitis (ie, improved global overall clinical scores, reduced exacerbations by 35% and hospitalisation by 44%).^35^ A short-term small (n=30) RCT in adults with radiographic bronchiectasis also found improved clinical outcomes (better cough and dyspnoea scores, 6 min walk test) on day 15.^36^ However, as with long-term macrolides to reduce acute exacerbations,^1 7^ such medications are usually given for prolonged periods and this 15-day study was too short to determine whether the observed clinical benefits were sustained.

The coexistence or overlap of COPD with bronchiectasis has long been recognised. A meta-analysis of 18 studies found that the prevalence of bronchiectasis in COPD was 54% (range 25%–69%).^37^ Another meta-analysis of 14 observational studies described comorbid bronchiectasis in COPD with increased the risk of exacerbation (1.97, 95% CI 1.29 to 3.00), severe airway obstruction (1.31, 95% CI 1.09 to 1.58) and mortality (1.96, 95% CI 1.04 to 3.70).^38^ This meta-analysis also reported a wide range of coexistent bronchiectasis from 4% to 72%.^38^ Consequently, studies involving erdosteine in adults with COPD are also relevant in informing the rationale for our study.

In adults with COPD, a systematic review of interventions for preventing respiratory exacerbations included two RCTs using erdosteine.^27^ Both RCTs (8–12 months duration) found that erdosteine significantly reduced hospitalisation, antibiotic use and respiratory exacerbations and improved QoL and lung function.^27^ A meta-analysis^28^ of individual patient data (n=1046) on erdosteine also found it was associated with significant symptom amelioration, compared with either placebo or other mucoactive agents in patients with chronic bronchitis/COPD. A network meta-analysis^39^ (seven RCTs) found that erdosteine was superior to other mucoactive agents (eg, carbocysteine) for reducing acute COPD respiratory exacerbations and hospitalisation. A more recent post hoc RCT analysis of adults with moderate-to-severe COPD also described clinical benefits when erdosteine was added to their usual maintenance therapy for a 12-month period (compared with placebo).^40^ The improved clinical outcomes included improved QoL and patient-reported disease severity and reduction in proportion of patients who had exacerbations requiring antibiotics and/or corticosteroids (74.3% vs 80.8%, p<0.05).^40^

Thus, erdosteine is a promising novel mucoactive that may be beneficial for people with bronchiectasis. It is possible, but unknown, if prolonged treatment for 12 months will benefit children and young adults with bronchiectasis. As these actions target part of the underlying pathobiological mechanisms in people with bronchiectasis, it is plausible that regular erdosteine could improve clinical outcomes.

### Aims and hypotheses

Our primary aim is to determine in children and adults aged 2–49 years with bronchiectasis whether regular erdosteine over a 12-month period reduces acute respiratory exacerbations compared with placebo. Our primary hypothesis is that people with bronchiectasis who regularly use erdosteine will have fewer exacerbations than those receiving placebo.

Our secondary aims are to determine the effect of the trial medications on QoL and other clinical outcomes (exacerbation duration, time-to-next exacerbation, hospitalisations, lung function, adverse events). Our secondary hypotheses are that regular erdosteine improves QoL and other clinical outcomes.

Additionally, we will also the assess cost-effectiveness of the intervention by collecting healthcare-related costs and calculate the incremental cost-effectiveness ratio of the active treatment. Our additional hypothesis is that regular erdosteine will be associated with less exacerbation-related healthcare resource utilisation and costs. Finally, there is a discovery aim to determine if blood gene expression and/or microbial biomarkers will identify pathways and/or predict those at greater risk of recurrent respiratory exacerbations.

## Methods and analysis

### Design

We are undertaking a parallel, double-blind, placebo, superiority multicentre RCT (with concealed allocation) to determine the efficacy of 12 months of erdosteine at reducing acute respiratory exacerbations and improving other clinical outcomes. We randomised the first participant in May 2021 and we anticipate continuing recruitment until at least July 2024. This RCT started recruiting concurrently with our other RCT that also involved erdosteine in one of its arms, where the goal was to reduce exacerbations in people with primary ciliary dyskinesia.^41^ Thus, these RCTs have many shared methods and descriptions of data collected.

### Study settings and participants

#### Study sites

Our current study sites are across three countries: Australia, Malaysia and the Philippines. The Australian sites are Queensland Children’s Hospital (Brisbane), Mater Hospital (Brisbane), Concord Repatriation Hospital (Sydney), Perth Children’s Hospital (Perth), Royal Darwin Hospital (Darwin) and Gold Coast University Hospital (Gold Coast). The Malaysian site is the University of Malaya Medical Centre (Kuala Lumpur) and in the Philippines, children are recruited from St Luke’s Hospital (Quezon City). Adults are recruited only from the Mater and Concord Repatriation Hospitals.

#### Inclusion criteria

Inclusion criteria are as follows: (1) Children or adults aged 2–49 years, (2) have radiographic-confirmed bronchiectasis with at least two exacerbations or one hospitalisation for an acute respiratory exacerbation in the last 18 months and (3) are able to be contacted for the 15-month follow-up period.

#### Exclusion criteria

Exclusion criteria are as follows: (1) CF, (2) contraindications for erdosteine (eg, liver dysfunction, hypersensitivity, renal failure, cystathionine-synthetase enzyme deficiency, phenylketonuria, active peptic ulcer disease), (3) pregnant, pregnancy planned (in the next 12 months) or nursing mothers, (4) previously randomised, (5) hospitalised in the last 4 weeks for a respiratory illness, (6) if another household member is a participant in this study or (7) active participation in another RCT.

#### Recruitment

Potential participants are identified from clinic lists and hospital databases and are approached by a research team member uninvolved in their clinical care. Those fulfilling the eligibility criteria and are willing to participate will have the study explained to them by a member of the research team using an information booklet, as well as providing consent documents and interpreters (where appropriate). After all aspects of the study are explained and agreed on, written informed or electronic consent (e-consent) is obtained from the parent/guardian/participant, including assent in children aged 12–17 years. Our processes are consistent with the principles of Good Clinical Practice (GCP), the Declaration of Helsinki and the Australian National Health and Medical Research Council (NHMRC) requirements and cultural aspects of consent for Indigenous Australians.

### Study protocol

**Figure 1:**
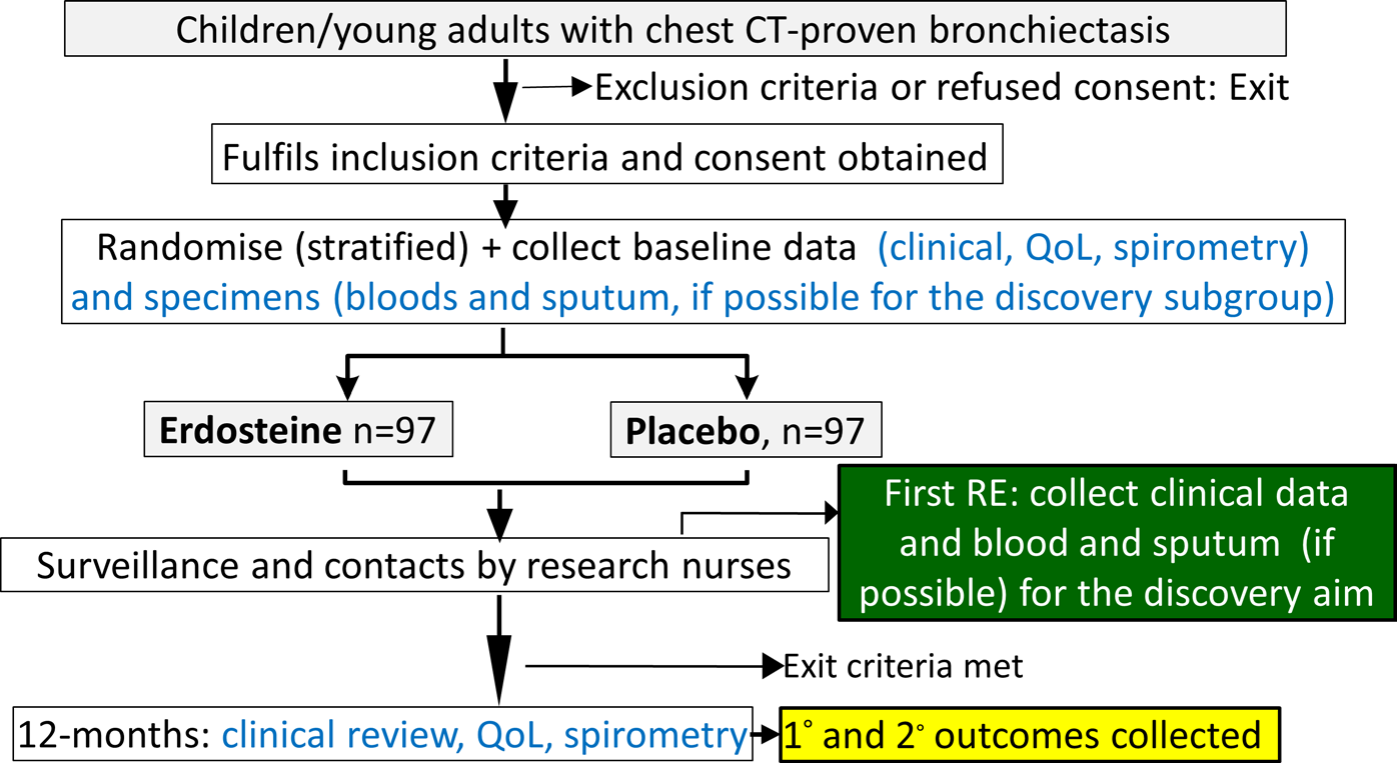
Overall schematic study design of our randomised controlled trial. QoL, quality of life; RE, respiratory exacerbation.

A summary of our protocol is summarised in the figure 1. All participants are managed according to local clinical protocols for their other bronchiectasis-related care.

#### Trial medications

Erdosteine is available as oral powder for suspension (175 mg/5 mL) or capsules (300 mg) administered two times per day for 12 months. Doses are weight based, that is, <15 kg: 2.5 mL/dose; 15–19 kg: 5 mL/dose; 20–30 kg: 7.5 mL/dose or one capsule; >30 kg: 10 mL/dose or one capsule. Equivalent placebo capsules or oral suspension also match the active preparation. We purchased erdosteine and its corresponding placebo from the manufacturers (Recipharm, Paderno Dugnano, Italy).

#### Randomisation, allocation concealment and blinding

Randomisation is undertaken by each study site’s pharmacy using REDCap where the sequence is stratified by site in permuted blocks sizes of 4–8. Allocation is thus undertaken by the local hospital pharmacist (or representative) who then dispenses the trial medications to the participant. Trial medications are dispensed every 3 months and adherence is monitored by return of bottles, collection of study medications and parent reports.

People who are blinded to treatment allocation are (1) receiving the treatment, (2) administering the treatment, (3) assessing the outcomes and (4) analysing the results/data.

#### Data and sample collection, monitoring and follow-up details

All collected data are recorded by GCP-trained researchers using standardised data sheets or online surveys (similar to our previous RCTs).^18 19 42^

At enrolment, sociodemographic/clinical data, spirometry (in those aged >6 years) and specimens (sputum and blood where possible in a subset of participants contributing to the discovery component) are collected (table 1).

**Table 1:** Schedule for visits and contact points

Visit type	Baseline	Month												
		1	2	3	4	5	6	7	8	9	10	11	12	Start of exacerbation (if possible)
Screening for eligibility	√													
Informed consent (paper or electronic)	√													
Randomisation	√													
Medical history	√													
Medical chart review	√			√			√			√			√	
Monthly parent/participant contact recording exacerbations		√	√	√	√	√	√	√	√	√	√	√	√	
Clinical assessment (where possible)	√			√			√			√			√	√
PC-QoL-and/or QoL-B questionnaire	√						√						√	√
Spirometry (if possible)	√			√			√			√			√	√
Urine pregnancy test (only females ≥14 years	√						√						√	
Study diary	√						√						√	√
Blood in subgroup	√													√
Sputum in subgroup	√													

B-QoL, bronchiectasis QoL tool; PC-QoL, Parent-proxy Cough QoL-8 instrument; QoL, quality of life.

Sociodemographic data include details of age, gender, family and household size, cigarette smoking, family and immunisation history, medications and comorbidities. These are obtained from the carers/participants and/or reviewing the medical notes. Participants are contacted every month via electronic communication (email or short-message service texting) or by telephone to monitor progress and adverse events (eg, vomiting, diarrhoea, rash) and economic data (eg, medical visits, treatments, other interventions, school/work absenteeism). Participants are seen in the clinic 3–4 monthly as part of their routine clinical care (if they reside locally at one of the study sites) and at the onset of their first exacerbation following enrolment where additional clinical data and biological specimens are collected (table 1), if possible. Specimens will be stored and analysed later. We have established robust methods for capturing respiratory exacerbations.^18 19 43^ Hospital records are also reviewed for hospitalisations. Additionally, at enrolment, a pregnancy test is undertaken if a female participant is aged ≥14 years.

Exit criteria include consent withdrawn, severe study-related adverse events, intolerance to study medications or any other clinical indication as determined by study site doctors, treating clinicians or the independent data monitoring committee (iDMC). Unblinding, if necessary, will be done by the site’s pharmacy

### Outcomes and endpoints

Our primary outcome is the acute respiratory exacerbation rate by 12 months postrandomisation. These are collected by participant reports (active surveillance of participants) and medical records.

In children, we will use the definition used previously^18 43^ that is also in accordance with the ERS-based guidelines^2^ and the ERS Task Force international consensus definition of an exacerbation.^3^ For children, an episode is defined by an acute respiratory event that (1) is treated with antibiotics and (2) has an increase in sputum volume and/or purulence, or >3 days of change in cough (at least 20% increase in cough score or change in quality (dry to wet/productive)) or physician-confirmed acute change in respiratory rate, effort or chest signs.^3^ For adults (aged>18 years), we will use the adult consensus definition that is, a clinician determines that treatment change is required and deterioration in ≥3 of the following for ≥48 hours (cough, sputum volume or purulence, breathlessness, exercise tolerance, fatigue and/or malaise, haemoptysis).^24^

Other outcomes (see online supplemental material for further information) are as follows:

10.1136/bmjresp-2023-002216.supp1 Supplementary data

- QoL scores at 12 months: In adults, we are using the bronchiectasis QoL tool (B-QoL),^44^ which has 37 questions with a respiratory subdomain score. In the absence of a bronchiectasis-specific QoL for children, we are using the validated Parent-proxy Cough QoL-8 instrument (PC-QoL).^45^
- Time-to-next exacerbation: Defined from the time of commencing trial medications to the next respiratory exacerbation (defined above), measured in days.
- Time-to-next respiratory-related hospitalisation.
- Spirometry values (forced expiratory volume in 1 s (FEV_1_ z-score) and forced vital capacity (FVC) z-score, and FEV_1_/FVC ratios).
- Adverse events, including gastrointestinal symptoms (eg, nausea, vomiting, abdominal pain, diarrhoea), headaches, rashes, use of additional non-macrolide antibiotics for non-pulmonary infections during the intervention, that is, for 12 months postrandomisation.
- Healthcare resource use.

### Data monitoring, management and analyses

All identifiable information on study participants will be retained in password-protected files stored on the QUT server or locked cabinets/rooms at study sites. Access to this information will only be provided to immediate study staff unless required by legislative or regulatory agencies and the HRECs. The principal investigator and statisticians will have access to the final trial dataset.

All adverse events, including serious adverse events, are monitored by an iDMC, which was established prior to commencing the study. An Indigenous (First Nations) Reference Group based at the Menzies School of Health Research (Darwin) oversees the cultural aspects of the study. Data coding and entry are coordinated in Brisbane and conducted in accordance with GCP.

The analyses will be directed by biostatisticians (Chatfield and Yerkovich). Results will be reported and presented in accordance with Consolidated Standards of Reporting Trials guidelines. We will use the ‘intention-to-treat’ approach for the main analyses. We also plan a ‘per-protocol’ analysis. Missing data will not be imputed. No interim analysis is planned. A detailed statistical analysis plan will be developed and approved by the chief investigators and the iDMC before undertaking the final analysis. Any exploratory, post hoc or unplanned analysis will be identified clearly. Analysis code will be written and agreed on knowledge of treatment allocation and adherence is provided to the statistician, as done previously.^18 43^

#### For the primary aim

To assess erdosteine efficacy, the exacerbation rate in those randomised to receive erdosteine will be compared with controls (receiving placebo). We will use a negative binomial regression model (as recommended,^46^ including treatment group, and number of months in the study included as an offset) to determine between-group differences (with 95% CI), as done previously.^19^

### For the secondary aims

The change (12 months minus baseline) in (1) respective domain scores of the QoL tools and (2) spirometry values (FEV_1_ %predicted, FVC %predicted and FEV_1_/FVC) between treatment arms will be analysed using ANCOVA (analysis of covariance) and presented as the mean difference (95% CI). A Kaplan-Meier curve will be constructed for each group (intervention vs respective controls) for the time-to-the next exacerbation and respiratory-related hospitalisation, and an HR (using a Cox regression model) will be performed and reported.^18 19^

#### For the cost-effectiveness aim

Healthcare resource use will be collected and summarised for each trial arm for inclusion in subsequent cost-effectiveness analyses making comparisons consistent with comparisons made in the primary analyses.

#### Sample size

Based on the primary outcome (exacerbation rate), we want to detect a significant difference between those allocated to erdosteine vs placebo (controls). We will use a negative binomial model (similar to our previous RCT),^19^ and we will have 90% power (alpha=0.05) to detect a 40% reduction in the respiratory exacerbation rate in the erdosteine group (controls=3/year, intervention=1.8/year), if we obtain final endpoint data from 164 persons. To account for a 25% attrition loss, we will enrol a total of 194 people. We conservatively estimated a 40% reduction in respiratory exacerbation rate based on the following: a paediatric study (total n=158) reported an absolute reduction in cough by 47% in those receiving erdosteine^32^ while adults with COPD allocated erdosteine (total n=155) had 58% fewer days in hospital,^27^ compared with placebo.

We postulate superiority between groups for QoL. The minimal clinical important difference for the PC-QoL instrument is 0.9^45^ (SD 1.8) while that of the adult B-QoL tool is domain dependent (7–10) (SD 17).^44^ With the standardisation of the scales, we will have 90% power to detect a difference of means between groups of 0.5 SDs. We are aware our study is insufficiently powered for the other secondary outcomes.

#### Trial oversight

The study is being monitored by an iDMC. The iDMC meets every 4–6 months; its frequency is deemed by the members. The trial is overseen by the chief investigators of the grant (listed below). It is sponsored and monitored by the Queensland University of Technology (Brisbane, Australia).

#### Patient and public involvement

The grant submission was informed by both patient and public consultation. The formal research team and the original successful Australian Medical Research Future Funds grant applicants include a parent of a child with bronchiectasis. The study design was presented to and discussed with our First Nations advisory group (https://crelungs.org.au/cre-first-nations-advisory-group) and parent and community advisory group (https://crelungs.org.au/cre-parent-and-community-advisory-group) prior to submission of our grants. After we obtained our grant, our knowledge transfer plan was codesigned with the assistance of both advisory groups. This RCT is also part of the suite of studies within our Centre of Research Excellence^47^ funded by the Australian NHMRC whereby parents and patients inform our translational research.

## Discussion

As few RCTs target people with bronchiectasis, current pulmonary management is largely extrapolated from CF-based studies.^2 16 17^ Given the different pathobiological mechanisms between these conditions, there is a risk that some therapies beneficial for people with CF might have little benefit, or worse be detrimental to people with bronchiectasis, as shown by dornase-alfa in those with idiopathic bronchiectasis.^7 48^ Our RCT tests a therapeutic agent based on a sound scientific rationale and addresses some of these unmet needs by employing a novel mucoactive agent (erdosteine).^30^

Our RCT focuses on children and young adults for several important reasons: (1) intervening in early disease may arrest disease progression^1 7 49^ and (2) to limit the heterogeneity of bronchiectasis (by not including older adults who can have different underlying aetiologies and more comorbidities). Importantly, radiographic bronchiectasis in children (likely also in young adults) is reversible if treated early, thereby avoiding the later progressive decline in lung function.^7 49 50^ In contrast, adults with bronchiectasis symptoms from childhood have worse disease and poorer prognosis (c.f. adult-onset bronchiectasis).^51^ Thus, focusing on a younger age group will likely provide a greater impact on reducing future disease burden.

### Choice of study outcomes

Choosing outcomes that are both valid and consumer-informed is important. Respiratory exacerbations and QoL as our main outcomes were chosen when developing our study plan as they were considered the most important from the consumer perspective.^13 52^ Also, these are two of the key core outcomes for children with bronchiectasis.^52^ Furthermore, the published literature shows respiratory exacerbations have major negative health impacts on people with underlying lung diseases. In children with bronchiectasis, respiratory exacerbations are particularly important clinically as they are associated with increased psychological stress, impaired QoL, lung function decline (−1.9 FEV_1_% predicted per hospitalised respiratory exacerbations) and substantial healthcare costs.^23 53^ Moreover, each child hospitalised in Brisbane, Australia with an acute exacerbation of their bronchiectasis cost the public health system in 2016 $A30 182 (US$22 407; £16 642 and €20 397).^53^ Thus, we chose respiratory exacerbation rate as our RCT’s primary endpoint.

Our other secondary outcomes (hospitalisation, lung function) are also important from both consumer and clinical perspectives, as voted on by parents of children with bronchiectasis.^2^ However, our sample size may have limited power to detect a significant difference between groups for these outcomes.

We used the standard definitions to identify acute exacerbations for determining our primary outcome, in line with current ERS guidelines for bronchiectasis,^2^ and for defining exacerbations^3^ in children. For adults, we used the adult consensus definition.^24^

### Potential study limitations

Although our study has many novel and robust aspects, there are also limitations. First, while including children and adults in a single RCT is a strength and addresses criticisms of children not being involved sufficiently in clinical trials,^54^ it may also be a limitation as children with bronchiectasis have many different characteristics to adults.^7 49^ Including a wide age range increases the heterogeneity of the target population. We have attempted to minimise this potential limitation by not recruiting older adults aged >50 years and stratifying randomisation by site since centres recruited only either children or adults, and not both. We plan to include age in a sensitivity analysis. Second, we developed our protocol before the international consensus on the core outcome set (COS) for children with bronchiectasis^52^ was developed. The COS arose from a systematic review and international surveys that had 562 respondents (201 parent/patients; 361 healthcare professionals) from 59 countries.^52^ As such our outcomes do not include two of the five COS items, namely unscheduled healthcare visits and symptoms. Also, although we included QoL tools, there is currently no validated bronchiectasis-specific QoL instrument for children. Third, our study is underpowered for some secondary outcomes like lung function and hospitalisations. Fourth, considering the adverse impact of the SARS-CoV-2 pandemic (see the ‘Impact of the SARS-CoV-2 pandemic upon the clinical trial’ section), our RCT may be underpowered for assessing the efficacy of erdosteine for the primary outcome too.

### Impact of the SARS-CoV-2 pandemic on the clinical trial

The recent SARS-CoV-2 pandemic affected several aspects of our RCT. First, in 2021–2022, our recruitment was severely affected by strict state and regional lockdowns and travel restrictions preventing face-to-face visits required by our study. Also, the reluctance of parents/patients to attend clinics during the pandemic prevented effective recruitment. However, recruitment recovered as restrictions were eased. Second, the incidence of exacerbations over this period was substantially lower than that predicted, a phenomenon found worldwide coincident with the public health measures enacted to reduce transmission of SARS-CoV-2.^55 56^ Third, there are also financial consequences as no additional funds were made available by the funding body and research team members continued to be paid throughout this pandemic.

To address these challenges, we initiated several mitigations steps that included (1) using e-consents (approved by all ethics committees); (2) posting trial medications to the participant’s home (instead of participants collecting trial medications from hospital pharmacies) and (3) undertaking online (telehealth) follow-up where possible and appropriate. Nevertheless, we are uncertain whether our study’s findings will be adversely impacted by the SARS-CoV-2 pandemic.

### Summary

In this protocol paper, we describe our double-blind, superiority RCT examining the clinical benefits of 12-month treatment with erdosteine for children and young adults with bronchiectasis. Our consumer-informed clinical trial addresses the paucity of RCTs in the field. The multicentre nature of our study increases the generalisability of the future findings of our RCT.

We also plan additional side studies that have not been described fully in this protocol paper. These include a trial-based economic evaluation and undertaking a novel discovery aim (in a subset of study participants) to determine if blood gene expression and/or microbial biomarkers will identify pathways and/or predict those at greater risk of recurrent respiratory exacerbations. These additional aims will be reported later and will not form part of our primary RCT data analysis.

## Dissemination

Our RCT is conducted in accordance with Australian, Malaysian and Philippines legislation and NHMRC guidelines for ethical conduct of Research, including that for Indigenous Australians.

We will publish the results in a major medical journal and share the outcomes with the academic and medical community, funding and relevant patient organisations, including the Darwin First Nations Reference Group. Authorship eligibility guidelines will be used. We will not use of professional writers. We currently do not have any plans to grant public access to the full protocol, participant-level dataset or statistical code.
