# Supplementary material for: Erdosteine in children and adults with bronchiectasis (BETTER trial): study protocol for a multicentre, double-blind, randomised controlled trial
Source: BMJ Open Respir Res. 2024 May 7;11(1):e002216. doi: 10.1136/bmjresp-2023-002216 (PMC11086403; doi:10.1136/bmjresp-2023-002216)
Supplement: Supplementary data [file bmjresp-2023-002216supp001.pdf]

SUPPLEMENT

Additional details on Methods

Confidentiality is being maintained in accordance with Human Research Ethics Committee (HREC) requirements and Good Clinical Practice. All identifiable information on study participants is retained in password protected files inside locked cabinets and/or locked rooms at the study sites. Access to this information is available only to immediate study staff, unless required by legislative or regulatory agencies and the HRECs. No identifying information will be included in study reports. Clinical specimens are labelled with the participant unique identification and corresponding laboratory numbers.

Quality-of-Life (QoL) measurements

We are using the validated Parent-proxy Cough QoL-8 instrument (PC-QoL),[1] and the shortened version of the 27-item tool we used previously in our bronchiectasis randomised controlled trials (RCT).[2,3] Both versions have a summary score of 1–7. The adult bronchiectasis QoL tool (B-QoL)[4] has 37 questions with a respiratory sub-domain score.

Spirometry

Spirometry is performed by trained personnel with study participants being in a seated position with a nose clip in place according to American Thoracic Society and European Respiratory Society guidelines.[5,6] Measurements are valid when maximum inhalation is followed by a rapid forced exhalation until a volume plateau is achieved (usually >3-seconds expiratory time when aged <10-years and >6-seconds when ≥10-years). Sites use their own spirometers. In the presence of airway obstruction, where possible post-bronchodilator spirometry is performed 10-minutes after 400 mcg of salbutamol is delivered through a spacer. The highest forced expiratory volume in 1-second and forced vital capacity values are reported after three valid measurements, post-salbutamol.

Adverse-events

While erdosteine is a novel drug for the purpose we are examining in our RCT, it is widely available in over 40 countries worldwide, including being over-the-counter (i.e. no prescription required) in some countries. Nevertheless, we are collecting data on adverse-events in our RCT as a secondary outcome. In light of the above, we do not anticipate any increase in clinically-important adverse-events attributable to erdosteine.

Consent form

An example of a consent form is provided in the next page.

MASTER - INSERT HOSPITAL NAME

Parent/Guardian Consent Form

**HREC Study Number:**	**HREC/20/QCHQ/64068**
**Research Study Title:**	**B**ronchi**E**ctasis **T**rial **T**esting **ER**dosteine
**Participant's Name:**	

By signing this form, I acknowledge that I have read and understood the Information Sheet for this study. I know that my child's participation in this study is voluntary, and **that I may say NO**.<br>I understand that I can withdraw my child from the study at any time, and this will not affect his/her access to the best treatment available from the **INSERT HOSPITAL NAME**	Yes ☐ No ☐
I understand that this research involves my child taking trial study medication for 12 months.<br>I understand that it could be either active medication or a placebo.<br>I give permission for my child's medical records to be reviewed for illness related to lung health.	Yes ☐ No ☐
If my child is female and ≥ 14 years, I consent to staff performing a urine pregnancy test at enrolment and every 6 months during the study.	N/A for males<br>Yes ☐ No ☐
I understand that follow-up is for 15 months, during which time I will be contacted monthly for 15 months to be asked about my child's lung health. I am expected to complete monthly cough diary questionnaires as well as a Quality of Life (QoL) questionnaire every 3 months (at enrolment and 3, 6, 9, 12 and 15 months after enrolment).	Yes ☐ No ☐
I understand that if possible, my child should attend clinic visits every 3 months during the 15 months of study duration (at enrolment and 3, 6, 9, 12 and 15 months after enrolment).	Yes ☐ No ☐
I give permission for the study team to share my mobile phone number with "Twilio", (a cloud communications platform) to enable surveys to be sent to me via SMS.	Yes ☐ No ☐
If I have any problems or queries about how the study is conducted, and I do not feel comfortable contacting the research staff, I know I may contact CHQ HREC (07) 3069 7002.<br>I have had an opportunity to ask questions and I am satisfied with the answers I have received.<br>I understand I will receive a copy of the Information Statement and Consent Form to keep.	Yes ☐ No ☐
I consent to my child's de-identified data being used in future studies by the researchers and by their colleagues.	Yes ☐ No ☐

**Parent/Guardian Name**	**Parent/Guardian Signature**	**Date**
**Witness Name** (witness required if Parent/Guardian is unable to read/understand information sheet without assistance)	**Witness Signature**	**Date**

<table>
<tr><td>Participant Name – as stated at top of page</td><td>Participant Signature (if deemed appropriate)</td><td>Date</td></tr>
<tr><td colspan="3">Research Staff Declaration: I have given a verbal explanation of the research project, its procedures, and risks. I believe that the parent/guardian has understood the explanation.</td></tr>
<tr><td>Researcher Name</td><td>Researcher Signature</td><td>Date</td></tr>
</table>

References

1 Newcombe PA, Sheffield JK, Chang AB. Parent cough-specific quality of life: Development and validation of a short form. J Allergy Clin Immunol 2013; 131(4):1069-1074.
2 Goyal V, Grimwood K, Ware RS et al. Efficacy of oral antibiotics for non-severe exacerbations of bronchiectasis in children (BEST 1): A multi-centre, double-blind, double-dummy, randomised placebo-controlled trial. Lancet Respir Med 2019; 7(9):791-801.
3 Goyal V, Grimwood K, Byrnes CA et al. Amoxicillin-clavulanate versus azithromycin for respiratory exacerbations in children with bronchiectasis (BEST-2): A multi-centre, double-blind, non-inferiority randomised controlled trial. Lancet 2018; 392(10154):1197-1206.
4 Quittner AL, O'Donnell AE, Salathe MA et al. Quality of Life Questionnaire-Bronchiectasis: final psychometric analyses and determination of minimal important difference scores. Thorax 2015; 70(1):12-20.
5 Miller MR, Hankinson J, Brusasco V et al. ATS/ERS Taskforce series: Standardisation of spirometry. Eur Respir J 2005; 26(2):319-338.
6 Graham BL, Steenbruggen I, Miller MR et al. Standardization of Spirometry 2019 Update. An Official American Thoracic Society and European Respiratory Society Technical Statement. Am J Respir Crit Care Med 2019; 200(8):e70-e88.
